# Meaning from movement and stillness: Signatures of coordination dynamics reveal infant agency

**DOI:** 10.1073/pnas.2306732120

**Published:** 2023-09-18

**Authors:** Aliza T. Sloan, Nancy Aaron Jones, J. A. Scott Kelso

**Affiliations:** ^a^Center for Complex Systems & Brain Sciences, Florida Atlantic University, Boca Raton, FL 33431; ^b^Intelligent Systems Research Centre, Ulster University, Derry~Londonderry BT48 7JL, N. Ireland

**Keywords:** agency, dynamical systems, cognitive development, coordination

## Abstract

How do human beings make sense of their relation to the world and realize their ability to effect change? Applying modern concepts and methods of coordination dynamics, we demonstrate that patterns of movement and coordination in 3 to 4-mo-olds may be used to identify states and behavioral phenotypes of emergent agency. By means of a complete coordinative analysis of baby and mobile motion and their interaction, we show that the emergence of agency can take the form of a punctuated self-organizing process, with meaning found both in movement and stillness.

## Significance

Revamping one of the earliest paradigms for the investigation of infant learning, and moving beyond reinforcement accounts, we show that the emergence of agency in infants can take the form of a bifurcation or phase transition in a dynamical system that spans the baby, the brain, and the environment. Individual infants navigate functional coupling with the world in different ways, suggesting that behavioral phenotypes of agentive discovery exist—and dynamics provides a means to identify them. This phenotyping method may be useful for identifying babies at risk.

This paper addresses a fundamental problem—the origins of agency—that great scientists have remarked upon, almost since the beginning of physics as we know it. The problem is so obvious that some may take it for granted, as gravity once was. Witness, however, Bohr (1) (1958): “A description of the internal function of an organism and its reaction to external stimuli requires the word *purposeful* (italics his) which is *foreign* (italics ours) to physics and chemistry…” Or Schrödinger (2) (1951) in his famous book *What is life?* asks: “If my body functions as a pure mechanism according to the Laws of Nature, what is this ‘I’?” Or even Newton (3) (1675) himself: “The power of life and will by which animals move their bodies with great and lasting force… demonstrate that there has to be other (undiscovered) laws of motion.” The present research goes to the very core of this issue using human infants as a test field. Primarily used to study infant motor development, learning and memory (4–8), we harness the so-called Mobile Conjugate Reinforcement (MCR) paradigm (4) as an experimental entry point to investigate the origins of agency. Infants begin the MCR procedure as disconnected observers, but when one of their feet is tethered to a mobile hanging above, some infants discover that they can make the mobile move. Anecdotal evidence suggests that causal powers are discovered in coordination (9), but up to now quantifiable signatures of such are wanting (10, 11). Conscious agency may, for instance, appear suddenly as an ‘aha!’ experience (12–14) or gradually, but only a complete dynamical analysis of the experimental arrangement can tell.

Increased kicking during tethered interaction is classically interpreted as evidence that infant leg movements are reinforced by mobile motion which is assumed to be inherently rewarding (4). Recently developed quantitative models of MCR indicate that there is more to this story beyond reinforcement (11, 15, 16). The present analysis, which includes measures of baby activity, mobile motion, and their interaction, reveals an entirely different picture. We provide evidence 1) that conscious agency can emerge as a phase transition in a coupled dynamical system that spans the infant and the environment; 2) that not only the infants’ active movement but also the pause structure (‘stillness’) between movements matters (i.e., is informationally meaningful); and 3) that after the baby is decoupled from the mobile s/he continues to kick, a strong indication that the baby is anticipating the sensory consequences of its own movements (17). Our results are not without practical implications: Individual infants are shown to navigate functional coupling with the world in different ways, suggesting a dynamic phenotyping method that may be useful for preventive care and early treatment of infants at risk.

**Fig. 1.**
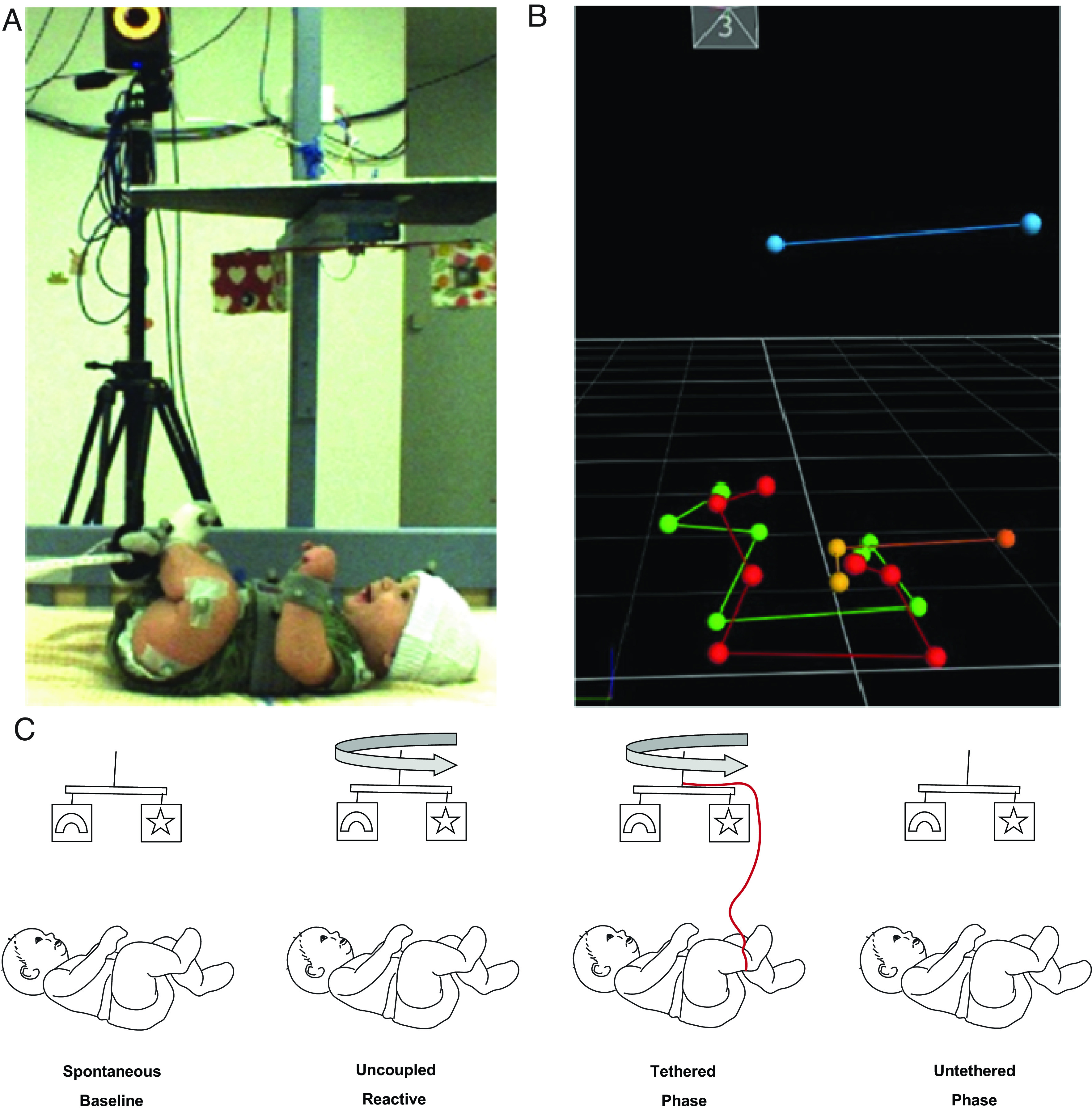
Experimental setup. (*A*) Infant and mobile are connected via ribbons and outfitted with spherical motion markers, (*B*) with marker positions represented in 3D space (infant’s left: red, right: green, torso/head: orange, mobile: blue). (*C*) The experimental paradigm proceeds from left to right (see text).

We tracked foot and mobile motion in 3D space at 100 Hz in 16 3 to 4-mo-old infants across four experimental phases: 1) A spontaneous baseline in which the mobile does not move. In the absence of stimulus motion, infant activity is spontaneously generated; 2) An uncoupled reactive phase in which the experimenter triggers mobile motion to measure the infant’s reaction to raw stimulation from a moving mobile; 3) A tethered phase which directly translates movements of the trigger foot (connected to a sensor by two ribbons) into mobile rotation rate; and 4) An untethered phase in which the ribbons are detached, and the mobile is again stationary, as in the baseline phase (Fig. 1).

How does coordination change as infants transition from being detached observers to active agents? We characterized multidimensional coordination between the feet and between each foot and the mobile for all infants across the experiment. As expected, the feet were more coordinated with each other (*r* = 0.56) than with the mobile when mobile motion was driven by the experimenter in the uncoupled reactive phase (trigger foot~mobile coordination: *r* = 0.24, *P* < 0.001; unconnected foot~mobile coordination: *r* = 0.20, *P* < 0.001). However, during tethering, trigger foot~mobile coordination (*r* = 0.73) was far stronger than either unconnected foot~mobile (*r* = 0.46, *P* = 0.001) or interfoot coordination (*r* = 0.52, *P* < 0.001), setting the coordinative stage for agentive discovery (Table 1).

**Table 1.** Coordination of 3D Velocity (*r* values)

	Spontaneous baseline		Uncoupled reactive phase		Tethered phase		Untethered phase	
Coordinative Pair	M	(SD)	M	(SD)	M	(SD)	M	(SD)
Trigger foot~Mobile	*na*	*na*	0.24	(04)	0.73	(0.11)	*na*	*na*
Unconnected foot~Mobile	*na*	*na*	0.20	(0.04)	0.46	(0.12)	*na*	*na*
Interfoot	.49	(0.13)	0.56	(0.13)	0.52	(0.13)	0.45	(0.15)

**Fig. 2.**
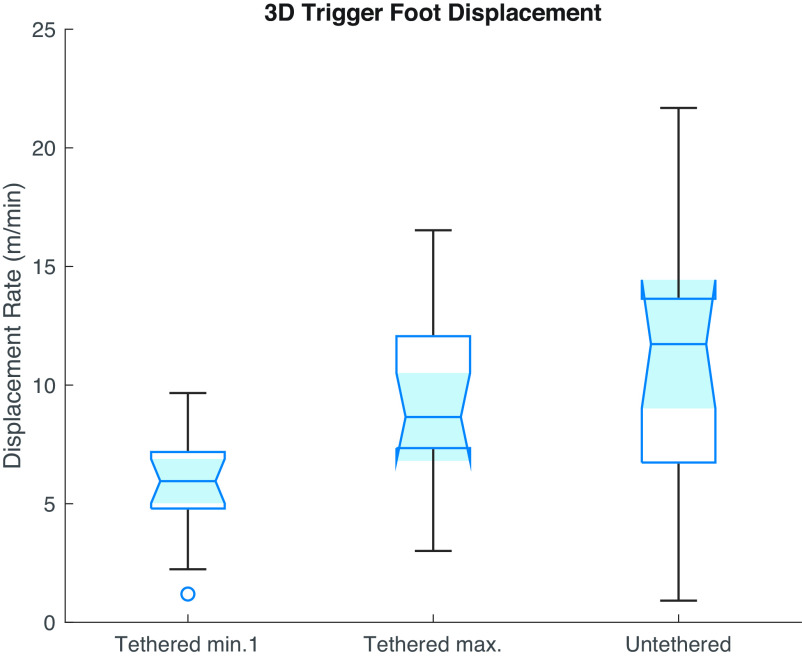
Activity after disconnection reflects infant expectation of mobile response. Distributions of trigger foot displacement rate are shown for the first minute of tethering, for each infant’s most active minute of tethering, and for the untethered phase. As expected, trigger foot displacement increased significantly on average across the tethered phase as infants interacted with the mobile. However, foot movement remained elevated even after the mobile and infant were disconnected, suggesting that infants are aware of their lost relationship to the mobile and attempt to recover it.

How does the amount of foot movement vary across conditions? Infants moved less on average when the mobile began moving in the uncoupled reactive phase compared to baseline (18). Foot movement dropped 34% when the experimenter triggered the mobile and remained suppressed in the first minute of tethering (*P* < 0.001), challenging the assumption that mobile motion simply rewards/stimulates kicking. On the other hand, infants moved significantly more during their most active minute of tethering (*M* = 10.12 m/min) relative to uncoupled reactivity (*M* = 6.07 m/min, *P* = 0.006) and the first minute of tethering (*M* = 6.42 m/min, *P* = 0.013). Notably, movement rate did not drop significantly from its peak after infants were untethered (*M* = 10.30 m, *P* = 0.33, Fig. 2), further challenging the idea that mobile activity reinforces infant activity. Instead, elevated movement rates after disconnection appear to reflect infant expectation of mobile response and attempts to reestablish the lost relationship (11, 19, 20). Further, peak tethered activity rate was more strongly correlated to infants’ activity in reaction to experimenter-triggered mobile movement (*r* = 0.64, *P* = 0.02) than to spontaneous rates (*r* = 0.46, *P* = 0.07), again underlining the importance of functional context for interpreting infant behavior.

**Fig. 3.**
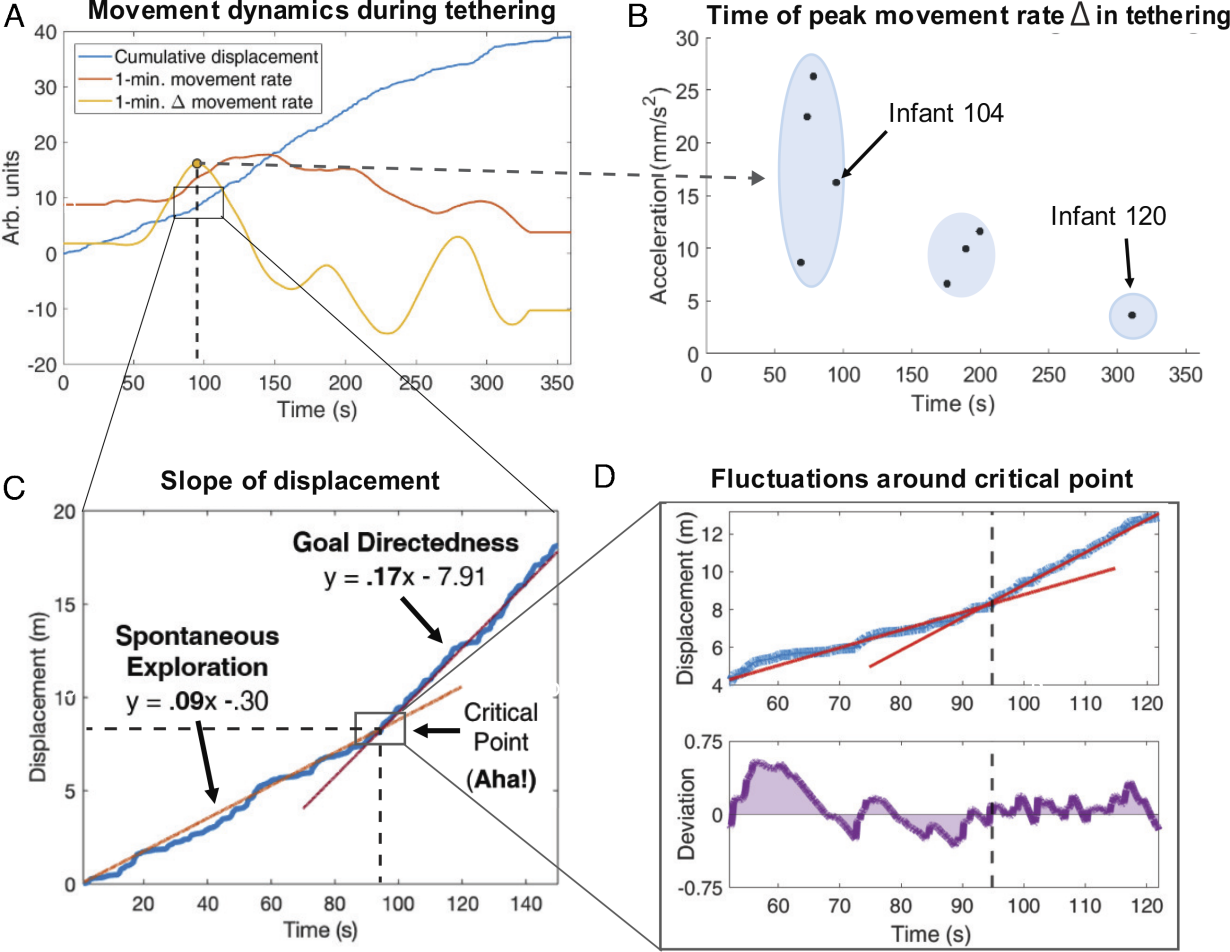
Individual phenotypes of emerging agency during tethered phase. (*A*) Tethered foot displacement (blue), movement rate (red), and change (Δ) in movement rate (yellow) for infant 104 (measures scaled to fit). To detect a moment of agentive discovery, we identified the peak Δ in movement rate (yellow dot). (*B*) Magnitude versus timing of peak Δ movement rate plotted for infants who increased activity ≥150% during tethering. Three distinct phenotypic patterns (blue ellipses) suggest different paths and timelines for agentive discovery. (*C*) A blow-up of (*A*) shows trigger foot cumulative displacement (blue) for infant 104 linearly modeled in the minute preceding and following peak Δ movement rate identified in (*A*) reveals a critical transition point at ~95 s. Slope of displacement nearly doubles as infant shifts from spontaneous exploration to goal-directed action, now purposefully triggering mobile movement. (*D*) A blow-up of (*C*) highlights change of slope at transition (*Top*; dashed line denoting time of transition). The lower panel shows that fluctuations are greater immediately before transition than after, a hallmark of (nonequilibrium) phase transitions (21, 22).

Infants clearly become more active during tethering, but what is the nature of change? Some infants might steadily increase movement rate during tethering, reflecting basic sensitivity to contingency, whereas others might realize their control over the mobile, suddenly increasing activity upon discovery (12). To capture the latter, we developed an “aha” detector by first differentiating trigger foot 3D displacement across 1-min windows to calculate tethered movement rate and differentiating again to locate each infant’s peak increase in movement rate (e.g., Fig. 3*A*). The greater the magnitude of this peak, the more abrupt the increase in movement rate. Although key findings are presented here, additional analyses regarding the validation of our methods are available in the results *SI Appendix*.

Three clusters of infants emerged: infants whose rate increase peaked early, midway, or late in tethering (Fig. 3*B*) with average rate increases of 281%, 175%, and 151%, respectively. Timing of peak rate increase was inversely related to magnitude of change and total increase across tethering. Using traditional methods, infants 104 and 120 would be identically classified as having met the standard criterion for contingency detection [i.e., 150% movement rate increase over baseline rate (4)]. However, unlike infant 104 who quickly discovered his causal abilities, doubling activity over just 1 min of tethering, infant 120 was still exploring her functional relationship with the mobile 6 min into tethering, slowly increasing activity throughout. Dynamics proves to be a critical tool for identifying moments of agentive discovery, differentiating agentive states and exposing underlying mechanisms. The finding of distinct clusters of infants suggests that behavioral phenotypes of agentive discovery exist—and that dynamics provide a means to identify them (see also *SI Appendix*, section 5).

**Fig. 4.**
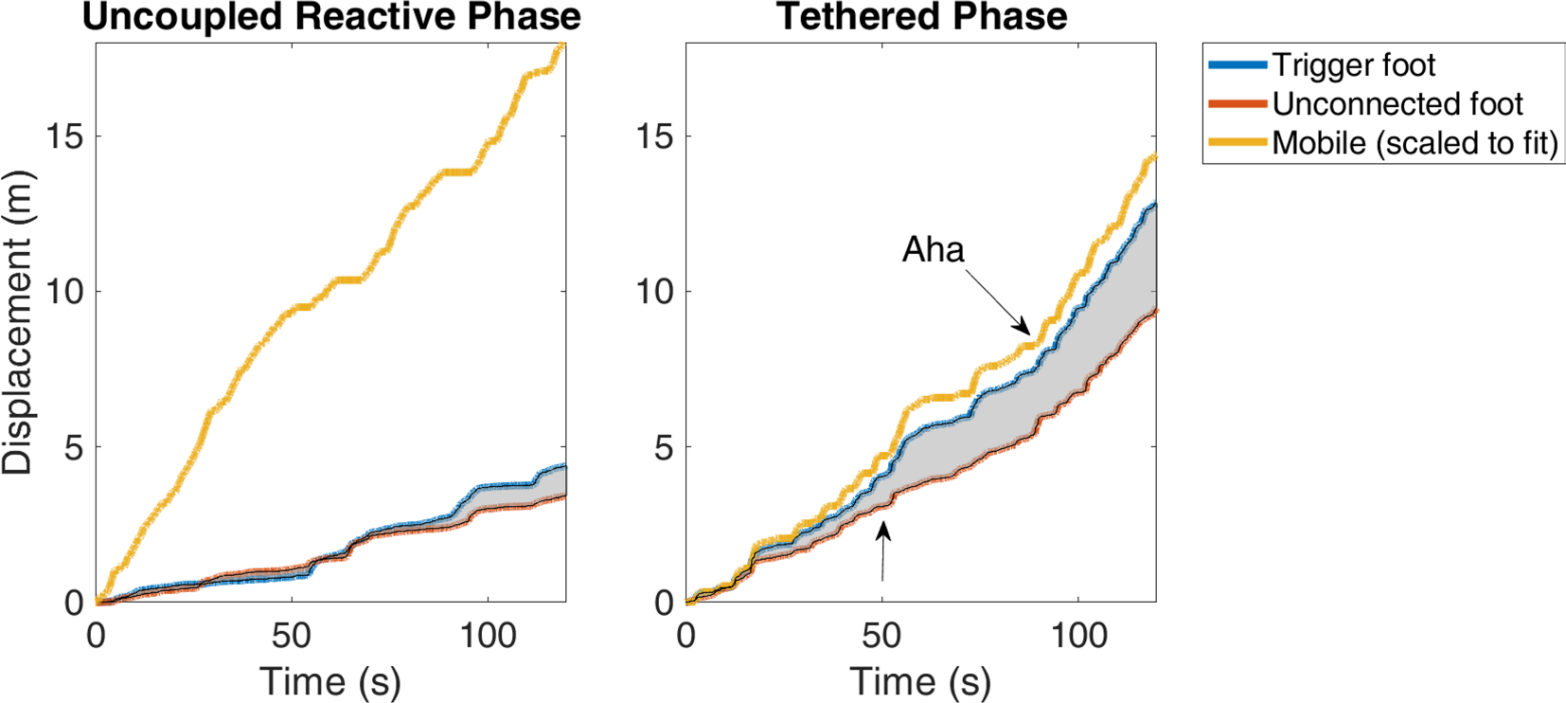
Changing coordination within the baby’s body and between baby and mobile precede discovery. Displacement of infant 104’s feet and the mobile demonstrate that the feet are more strongly related to each other than with the mobile before tethering, i.e., uncoupled reactive phase (*Left*), whereas during tethering (*Right*), the trigger foot and mobile are tightly coupled, and the relationship between the feet (shaded area) loosens after ~50 s.

To explore one form of phenotypic expression, infant 104’s journey to agentive discovery was analyzed in detail (Fig. 3 *A*, *C*, and *D*). When quantified as a change in slope of displacement, the activity burst indicating agency’s emergence resembles the sudden transitions seen in adult studies of sensorimotor coordination and learning (21, 22)(Fig. 3*C*). Differentiating agentive states is a prerequisite to understanding rules for transitioning between them. The magnitude of deviations from the foot displacement regression line is larger before the moment of discovery than after (Fig. 3*D*). Whereas phase transitions (state changes involving threshold crossing) in complex physical systems are typically anticipated by growing instability (critical fluctuations) and longer recovery times (critical slowing down), the magnitude of fluctuations in this infant progressively shrank. This unexpected reduction of fluctuations and critical speeding up just prior to transition has a functional interpretation aligned with teleological accounts in active learning (23–25): it reflects a decrease in exploratory behavior and an apparent increasing certainty of becoming a mindful body (26). Critically, infant 104’s realization of agency occurred within the context of tight trigger foot~mobile coordination and after disruption to the unconnected foot~mobile relationship (compare Fig. 4 *A* and *B*). When a strong, new relationship formed between the infant’s tethered limb and the mobile, a less functionally relevant relation within the infant’s body destabilized (Fig. 4*B*, shaded region).

It is essential to clarify that we found differences in fluctuation patterns surrounding the critical point among infants. Some infants displayed the more traditional indicators of criticality, namely greater fluctuations prior to the critical point, whereas the opposite was true for others (*SI Appendix*, Fig. S2). Reduced fluctuations after the critical point suggest enhanced control. In contrast, growing variability suggests increased exploration and/or play. Additionally, although most infants who exceeded the cutoff maintained their baseline foot preference during tethering, they showed a clear loosening of coordination between the feet surrounding the critical point (*SI Appendix*, Fig. S3). In contrast, coordination between the feet held steady or changed linearly in the babies that did not meet the 150% rate increase cutoff. Therefore, detection of functional relationship between the mobile and the feet in general may be preceded by or result in a loosening of this less functionally relevant coordinative relation within the body. To reiterate, the results presented for infant 104 depict just one of multiple dynamical pathways for the emergence of agency (*SI Appendix*).

**Fig. 5.**
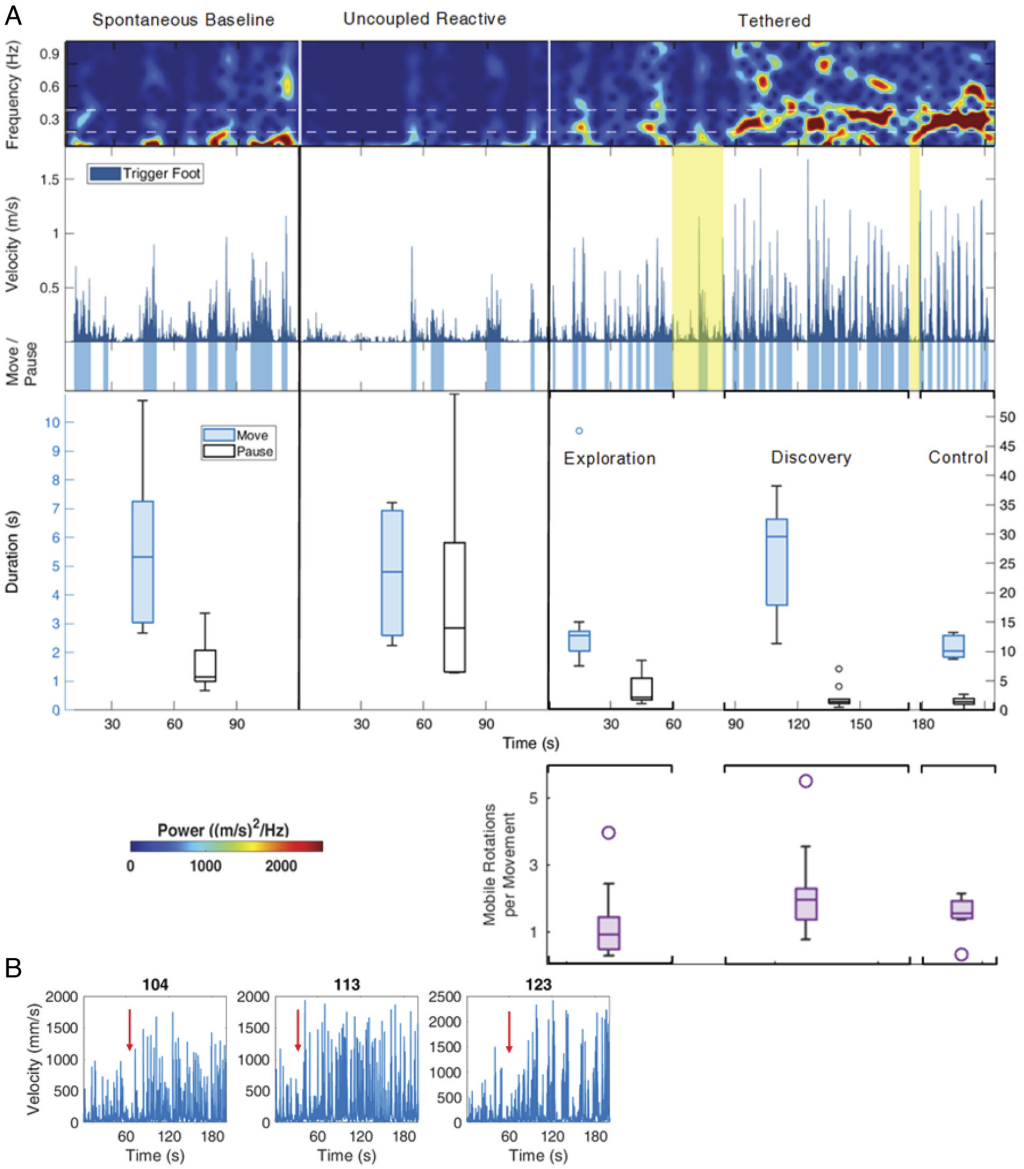
Agentive discovery is a punctuated process. (*A*) Foot movement is depicted from top to bottom in terms of spectral power, velocity (dark blue), duration of movement episodes (light blue bars) and pauses (white spaces), and distribution of duration of movements and pauses (blue and white filled boxplots, respectively). The purple boxplots show the number of mobile rotations elicited by each episode of infant movement across tethering. Lulls in activity during tethering from 60 to 85s and from 170 to 180 s (yellow highlights) precede hypothesized change: from exploration (0 to 60 s) to discovery (85 to 170 s, note elevated but variable infant and mobile activity in boxplots) to enhanced infant control [180 to 215 s, note consistent high-velocity foot activity and stable, elevated mobile response (purple boxplot)]. (*B*) For several infants, a period of relative rest (arrow) precedes a burst of trigger foot activity indicative of sudden discovery.

The influence of mobile motion on infant movement reveals further insight into the mechanisms of agentive discovery. In the first phase of the experiment (with no mobile movement), the infant spontaneously produced meandering movements each lasting 3 to 11 s (Fig. 5*A*, blue boxplot). However, whenever the experimenter triggered mobile rotation during the uncoupled reactive phase, the baby froze, leading to overall suppression of activity [note increase in pause duration (white boxplot)]. Upon tethering, infant 104 began to move as before, but because foot movement now triggered mobile motion, he immediately froze again, truncating his movement, and did not recommence until the mobile stopped. Alternation between abrupt action (*M* = 3.2 s) and reactionary freezing (*M* = 3.5 s) across the first minute of tethering produced a unique staccato movement pattern (reflected in the emerging dominance of 0.3 Hz activity) and created many opportunities for the infant to explore effects of both self-movement and inactivity. This staccato movement pattern was common across infants during the tethered phase (median movement duration = 3.21 s and median pause duration = 2.32 s, see *SI Appendix*, Fig. S4), strongly suggesting that infants probe their relationship to the mobile through short bouts of action and inactivity. Following a lengthy lull (60 to 85 s, highlighted yellow), infant activity spiked. Foot movement became nearly continuous with highly variable velocity peaks which elicited elevated but variable rates of mobile rotation, reflecting discovery. Thus, agentive realization appears to be directed by two opposing forces during tethering: an intrinsic drive to explore accompanied by a freezing response to novel mobile movement. Whereas episodes of movement communicate the degree of relatedness between infant and mobile, periods of inactivity afford crucial counterevidence. If the infant moved ceaselessly, there would be no way to rule out the possibility that some external force drives the mobile. Information about agency exists both in stillness and movement. For several babies, a sudden increase in trigger foot activity during tethering (i.e., agentive realization) was preceded by a brief period of relative rest (Fig. 5*B*, arrows). After a short pause at 170 s in tethering (highlighted yellow in Fig. 5*A*), infant 104 further discovered how to reliably elicit ~two mobile rotations per bout of movement with short duration, high-velocity foot strikes, precisely controlling his own motion and that of the mobile. Such discoveries are made in the gaps.

## Discussion

Previous MCR studies collapsed environmental stimulus and organismic response. Now giving each aspect its due, we find that the emergence of agency can take the form of a bifurcation or phase transition in a dynamical system (15): from a less correlated state to a state where both movements of mobile and the tethered limb are highly coordinated. Departing from classical reinforcement frameworks, the present analysis of coordination dynamics shows that the emergence of agency is a punctuated, self-organizing process (12) with meaning found both in movement and stillness (26). That the mobile moves when the baby moves but not when the baby pauses—like Cezanne’s pauses between brush strokes—confirms to the baby that ‘I can make things happen.’

## Materials and Methods

### Recruitment.

Birth records were obtained from the Florida Department of Health. Infants were recruited by postcards which were sent to all households within a 1-h drive of the university lab who had full-term (>36 wk), healthy (Apgar >7) infants under 3 mo of age. Infant gestational age was obtained from parents before testing to confirm that infants were full term. Informed consent was also obtained from parents before testing. This study was approved by the Florida Atlantic University (FAU) Social, Behavioral and Educational Research Internal Review Board and the Florida Department of Health Internal Review Board. The experiment was performed in accordance with FAU and Florida DOH guidelines and regulations. Informed consent from a parent and/or legal guardian was obtained for all infant participants prior to testing. Consent for photo release of infant participants was obtained from a parent and/or legal guardian.

### Demographics.

Sixteen 3 to 4-mo-old full-term infants (11 male and 5 female) participated in this study. Infants were on average 100.81 d old (*SD* = 16.29 d). Using WHO growth charts, these infants were on average at the 60th percentile for weight given their age (*SD* = 22.52%). INFANIB assessments found no significant motor development delays.

### Apparatus.

The current study measured infant and mobile movement in three-dimensional space using Vicon motion capture technology which employs eight infrared cameras. The system was set up to capture marker positions (distance from origin of the 3D space) at a rate of 100 Hz. Infants interacted with a feedback system which translated infant leg movements into mobile rotation. The apparatus consisted of a wooden arm with a pivot joint at its midpoint suspended above the crib parallel to the length of the crib. This arm was easily rotated in one plane only. An Adafruit BNO055 absolute orientation sensor, equipped with a nine degree-of-freedom accelerometer, magnetometer, and gyroscope, was attached to one end of the rotating arm suspended above the crib. This side of the arm was also very slightly weighted so that at rest the end with the sensor pointed downward. Two ribbons were tied to the other end of the arm. When these ribbons are pulled downward, the arm rotates and the sensor rises upward. The other ends of the ribbons were snapped onto a sock placed on the infant’s foot. Euler angles of the arm were measured by the sensor and transmitted to an Arduino Uno board which calculated the change in angle from cycle to cycle. If the change in Euler angle was greater than 1°, the Arduino sent a signal to a DC motor to begin spinning. A simple mobile consisting of a wooden plank with colorful cubes on either end was attached to the rotating shaft of the DC motor and was suspended above the infant’s face. The magnitude of the change in angle of the sensor was mapped onto the speed of the motor, meaning that a greater change in angle (associated with a leg movement of greater amplitude and force) produced faster mobile rotation. When the change in Euler angle was less than one degree, the motor was not activated. After measuring the angular change of the bar at the end of movements, the one-deg. threshold was chosen to be just high enough to eliminate the effects of the bar’s postkick wobble on the movement of the mobile. A piece of foam board was placed above the mobile to both focus the infant’s attention on the mobile and block the movement of the rotating arm outfitted with the accelerometer from the infant’s view.

### Procedure.

The experiment took place in the Human Brain and Behavior Laboratory at FAU. During the lab visit, the infant was outfitted with socks and reflective markers and then placed supine into a crib. The MCR procedure involved four phases: spontaneous baseline (no mobile movement), an uncoupled reactive phase (experimenter triggered mobile movement), tethered phase (tethered foot triggered mobile response), and untethered phase (tethered foot is disconnected and mobile is stationary). Each phase lasted 2 min, except for the tethered phase, which lasted up to 6 min. If an infant began to cry and could not be quickly soothed, the current phase of the experiment was ended prematurely. On average, the time lengths for each phase were spontaneous baseline — 101.53 s (*SD* = 42.94), uncoupled reactive phase — 97.46 s (*SD* = 44.72 s), tethered phase — 274.00 s (*SD* = 119.15), and untethered phase — 95.80 s (*SD* = 43.21 s). During the uncoupled reactive phase, care was taken to not coordinate mobile motion with infant motion.

### Data Preparation.

#### 3D velocity and displacement.

Velocity proves to be a significant control variable for the human brain (27). The first derivative of the position (velocity) for each of the three axes was calculated using the central difference method (28):

[1] $$v_{a,n}=\frac{x_{n+1}-x_{n-1}}{2T},$$

where *a* is equal to the axis, and *T* is equal to the sampling period (1/100 s). v is a 3 × *n* matrix. The magnitude of the 3D velocity is equal to the square root of the sum of the squared velocity matrix. The magnitude of the 3D velocity was calculated in this way for each foot. Three-dimensional displacement was calculated by cumulatively summing the 3D velocity.

#### Mobile rotation and epochs of mobile movement.

For any episode of infant movement, the starting position of the mobile is arbitrary. Furthermore, while the built-in Matlab function atan2.m can be used to convert position to angle, each time the mobile passes the point on its orbit which corresponds to 0 radians, atan2 inserts an artificial jump of 2π radians to signify the beginning of the next cycle. Mobile X and Y positions were transformed using trigonometric properties to calculate rotational angle of the mobile, ranging from 0 to π radians. Mobile angle reset to 0 each time the mobile stopped moving. However, the pseudoactivity from the reset did not contribute to the present data analysis and was removed for more accurate visualization. Dividing the cumulative sum of the absolute change in angle by two across an interval provides the number of rotations in that time period. (Dividing by two is necessary as 2π radians = 1 rotation).

A filter was constructed to investigate whether the length of time that the infants kept the mobile in motion changed across the tethered phase. The filter identified bouts of mobile motion when mobile velocity exceeded 30 mm/s for at least 150 ms. Mobile epochs were defined as bouts of mobile motion that resulted in at least a quarter of a full rotation. Epoch start and stop times were then retained for further analysis.

#### 3D movement dynamics within the tethered phase.

While it was predicted that infant activity would increase across the tethered phase, it was also logical to suppose that each infant would peak in activity at different time points during the tethered phase due to individual differences. Using a MATLAB function, movingslope.m (29), a 1-min-wide moving window was applied to the cumulative displacement curve of the trigger foot to estimate its rate of displacement (mm/s) across the Tethered phase using a locally linear approximation. The length of the moving window (1 min) matches the window length used in classic infant contingency studies (4). If multiple maxima were found to exist, the timing of the earliest maximum was retained as it represents the point when the infant first reached its maximum rate. These data were used to identify the start time of the most active minute and the maximum rate of displacement for each infant during the tethered phase. The tethered phase peak rate does not represent a set point in time during the tethered phase across infants. Rather, it is a measure of activity across a time window comparable to other time samples (1 min) reflecting maximum activity during the tethered phase across infants. This step allowed peak rate to be compared to movement rates of other experimental phases. The MATLAB function was reapplied to the displacement rate output (1-min velocity) to calculate acceleration (mm/s^2^) in 1-min windows. Peak acceleration is equivalent to the greatest change in rate of activity across 1 min of the tethered phase. Acceleration values represent whether changes in activity during the tethered phase occur abruptly or gradually.

#### Coordination dynamics.

Coordination between each infants’ feet and between each foot and the mobile was calculated. Standard methods of coordination analysis in signal processing are cross-correlation and cross-covariance. Fisher transforms of normalized cross-covariance values produce Pearson product moment coefficients which can then be statistically compared (30). Here, the magnitudes of various multidimensional velocity vectors (i.e., between the two feet, between the trigger foot and the mobile, and between the unconnected foot and mobile) were cross-covaried across the first minute of each phase for each infant using the aforementioned method to obtain Pearson r values. Differences between experimental phases were tested using repeated measures ANOVAs.

## Supplementary Material

Appendix 01 (PDF)

## Data Availability

Anonymized three-dimensional movement data and Matlab scripts have been deposited in OSF (https://osf.io/y82a9/?view_only=a8b6480789e84c5bb0d87aad8f95b2a7) (31). Video recordings of infants are not de-identified and, therefore, cannot be shared freely. Video recordings of study sessions are stored securely in accordance with IRB rules and regulations.
