# Supplementary material for: Meaning from movement and stillness: Signatures of coordination dynamics reveal infant agency
Source: Proc Natl Acad Sci U S A. 2023 Sep 18;120(39):e2306732120. doi: 10.1073/pnas.2306732120 (PMC10523583; doi:10.1073/pnas.2306732120)
Supplement: Supplementary file 1 — Appendix 01 (PDF) [file pnas.2306732120.sapp.pdf]

PNAS

Supporting Information for

Meaning from Movement and Stillness: Signatures of Coordination Dynamics reveal infant agency.

Authors:

Aliza T. Sloan[1], Nancy Aaron Jones[1], J. A. Scott Kelso[1,2]

Affiliations:

[1]Center for Complex Systems & Brain Sciences, Florida Atlantic University, FL, USA.

[2]Intelligent Systems Research Centre, Ulster University, Derry~Londonderry, N. Ireland

* Aliza T. Sloan.

Email: asloan2014@fau.edu

This PDF file includes:

SI Appendix – Results Supplement

Figures S1 to S5

SI Appendix – Results Supplement

Quantitative determination of critical points.

We developed a novel dynamical method to detect emergence of agency by determining the peak movement rate increase (*i.e.*, the critical point) and analyzing the difference in movement/coordination characteristics before and after this time. As exploration, agentive discovery and enhancement of causal control during tethered interaction are processes which unfold over time within individualized time frames, the analyses presented in the main body of the paper focused heavily on a single infant. The following Results Supplement quantitatively demonstrate how well the critical points are determined and explore whether common patterns of fluctuation and coordination change surrounding the critical point exist across infants.

1. Peaks in movement rate increase for all infants

Across all infants, each infant produced 1-4 acceleration peaks each (M = 2.38, SD = 0.72). Number of peaks did not differ among infants who exceeded or failed to exceed the 150% activity increase cut-off (p = 0.17). The maximum value of movement rate increase was chosen as the potential critical point for each infant. Some infants (*e.g.*, 120, 125) do not have clearly defined peaks, and the peak magnitude for these infants is near zero (see Fig. S1). This means that although the infants are moving and increasing their movement rate, they do so steadily, suggesting no sudden moment of insight. Others, such as 124 have peaks but do not meet the usual criterion of 150% increase. Momentary change in movement rate might produce a large acceleration peak, but without the overall 150% increase in movement rate that acceleration peak may give a false indication of infant discovery. The key point is that to convincingly claim infant agency, our method requires both the classical criterion to be met and the presence of essential dynamical features.

2. Fluctuations surrounding the critical point

To quantitatively characterize critical point determination, we performed regression analysis on displacement in a window of time before and after the acceleration peaks for each infant. Initially, we tried using a 1-minute pre- and post-peak window, but since some infants peaked close to either end of the tethered phase, this was not possible. The largest time window which could be applied to all infants was 45s before and after the critical point. Linear fit of displacement 45s before and after the critical point was strong, $R^2_{\text{pre-critical}}$ = 0.97, $R^2_{\text{post-critical}}$ = 0.99 averaged across all 16 infants. It appears from visual inspection of the data that different infants operate on different time scales both in terms of when they peak and in terms of the window size that best captures the shift surrounding the critical point. For some, a smaller time window surrounding the critical point better highlights dynamic switching. Using 20s windows before and after the critical point, the fit remains strong, $R^2_{\text{pre-critical}}$ = 0.95, $R^2_{\text{post-critical}}$ = 0.97 averaged across all 16 infants.

We found differences in fluctuation patterns surrounding the critical point. Some infants displayed the more traditional indicators of criticality, namely greater fluctuations prior to the critical point (*e.g.*, 104 and 117), whereas the opposite was true for others (*e.g.*, 113, 121, 123) (see Fig. S2). Reduced fluctuations after the critical point suggest enhanced control. In contrast, growing variability suggests increased exploration and/or play.

3. Coordination surrounding the critical point.

To explore critical dynamics of coordination, we plotted the cumulative displacement of the trigger foot (blue), the unconnected foot (red) and the mobile scaled to fit (yellow) throughout the reactive baseline (left panel) and in the time surrounding the critical point in the tethered phase (right panel, dotted vertical line) (see Fig. S3). Infants in the blue box exceeded the 150% cut-off, whereas infants in the red did not. Most infants who exceeded the cut-off maintained their baseline foot preference during tethering regardless of which foot was tethered to the mobile, suggesting that they detected a general relationship between the activity of mobile and their feet. We designed our feedback system to be extremely sensitive so that even infants with low activity levels could have the opportunity to interact with the mobile. Small postural adjustments of the tethered foot during untethered foot movement are sufficient to elicit a small response from the mobile. This may have obscured the unique relationship between the mobile and trigger foot for some infants, particularly those who had not matured to the point of producing unilateral leg movements (a capability that develops over the first few months). Note, however, that while displacement may have been greater for the unconnected foot than for the trigger foot during tethered interaction, coordination between mobile and the tethered foot was nonetheless stronger compared to the mobile and the unconnected foot (Table 1). Irrespective of differential leg displacement, these infants showed clear loosening of coordination between the feet (shaded in grey) surrounding the critical point. Therefore, detection of functional relationship may be preceded by or result in a loosening of less functionally relevant coordination within the body. In contrast, coordination between the feet held steady or changed linearly in the babies that did not meet the 150% rate increase cut-off.

4. Move & Pause Distributions.

To explore changes in the move and pause distributions, we computed movement and pause durations for each infant. To this end, each infant's 3D velocity data was smoothed using a Gaussian-weighted moving average over 1s windows. Next, epochs of movement were classified as velocity exceeding 50 mm/s for at least 150 ms. Pauses smaller than 50 ms were ignored. Movement and pause durations were aggregated across all infants for each experimental phase. The distributions for movement and pause durations are plotted in Fig. S4.

Movements were significantly shorter during the tethered phase compared to all other phases ($p < 0.01$). Paralleling patterns observed for infant 104 (Fig. 5), pauses elongated during the uncoupled reactive phase across infants ($p < 0.02$). Tethered phase pauses were also significantly shorter than the uncoupled reactive phase ($p < 0.001$). Although there is a visible constriction of pause distributions during tethering and in the untethered phase, pausing was not significantly shorter in either of these phases compared to the spontaneous baseline at the group level ($p_{tethered} = 0.26$, $p_{untethered} = 0.64$). The group level analyses performed here collapse behavior across tethering. In contrast, the patterns we observed in the individual analysis (say of infant 104 in Fig. 5) hinge on our dissection of the data across time. This temporal partitioning relies on the assumption that exploration, discovery and enhancement of control are processes which unfold in time during interaction. Collapsing data across time masks such dynamical features. Nonetheless, the fact that the tethered phase is dominated by short (~3s) movement epochs is highly suggestive of infants probing their relationship to the mobile. Overall, infants appear to actively explore their functional connection to the world through alternating short bouts of movement and inactivity.

5. Peak clusters

We plotted the acceleration peaks for all infants (see Fig. S5). Infants are separated into two groups: those who exceeded the activity cut-off (in black) and those who did not (in red). The original clusters (shaded in blue; see Fig. 3b) are largely preserved. Each infant is on its own

journey of exploration and discovery. Unlike standard methods which tally activity in distinct 1-minute windows, the sliding windows we use allow for every temporal possibility. Considering that there are 6 minutes of tethered interaction and infants might peak at any moment, this distribution in time (more than half of the infants peaking between 60 and 90 sec.) seems unlikely to arise from chance. Silhouette analysis of k-means for 1 to 6 clusters determined that there were 3 groups when applied to the 8 infants who met the movement threshold (silhouette criterion value = 0.98) and 2 groups when applied to all 16 infants (silhouette criterion value = 0.90). In general, criterion values range from -1 to 1, with values at these extremes indicating that clusters are far away from neighboring clusters. Though restricted by sample size our tentative clusters are therefore strongly defined. While the exact distribution of peaks will be borne out with further research, the fact that the two threshold groups largely overlap in the same temporal spaces supports the claim that much can be revealed by analyzing dynamics as opposed to counting kicks and imposing somewhat arbitrary activity cut-offs.

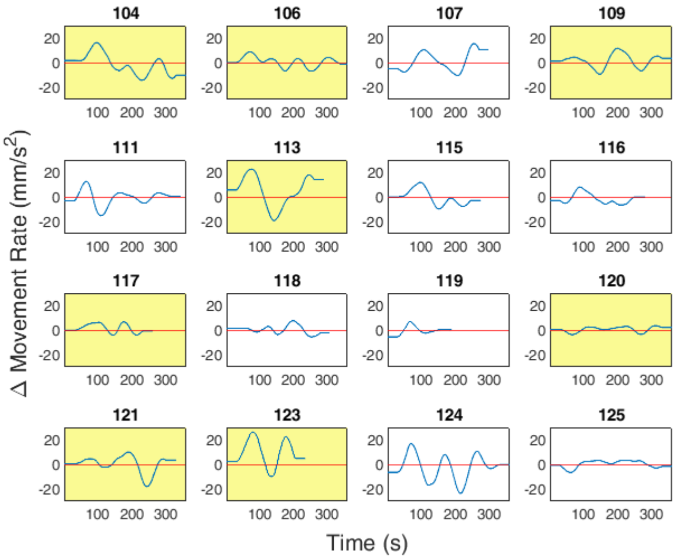

Fig. S1. Change in movement rate across the tethered phase. Change in movement rate is plotted for each infant with those who exceeded the 150% movement rate increase criterion highlighted in yellow. Peak change in movement rate represents the greatest increase in movement rate from one minute to the next during the tethered phase.

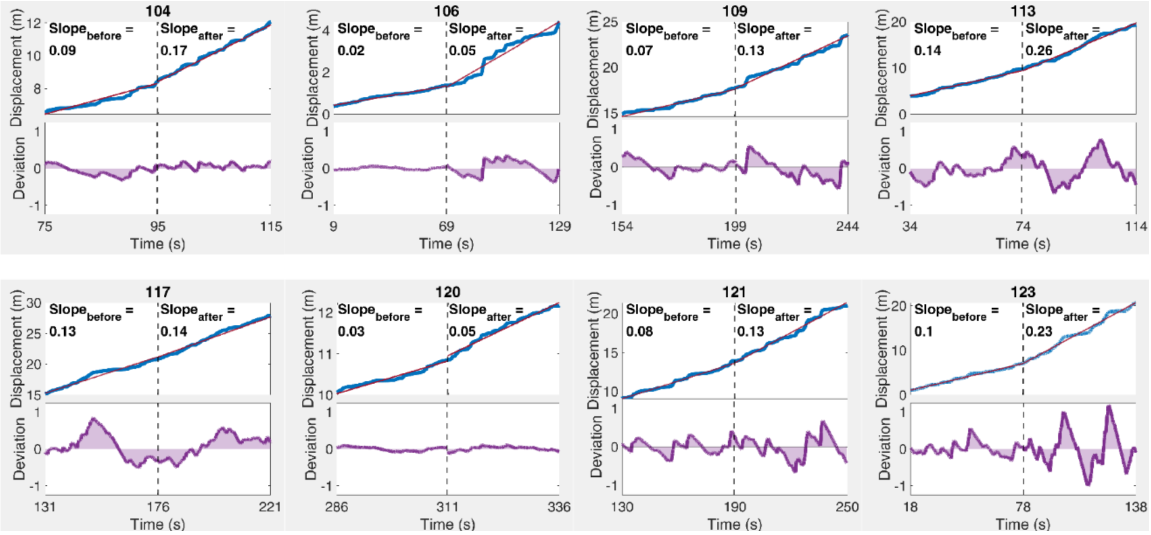

Fig. S2. Fluctuations around critical point. Change of slope is plotted at transition for each infant that met the 150% rate increase cut-off (top panel; dashed line denoting time of transition). Lower panels show fluctuations surrounding transition.

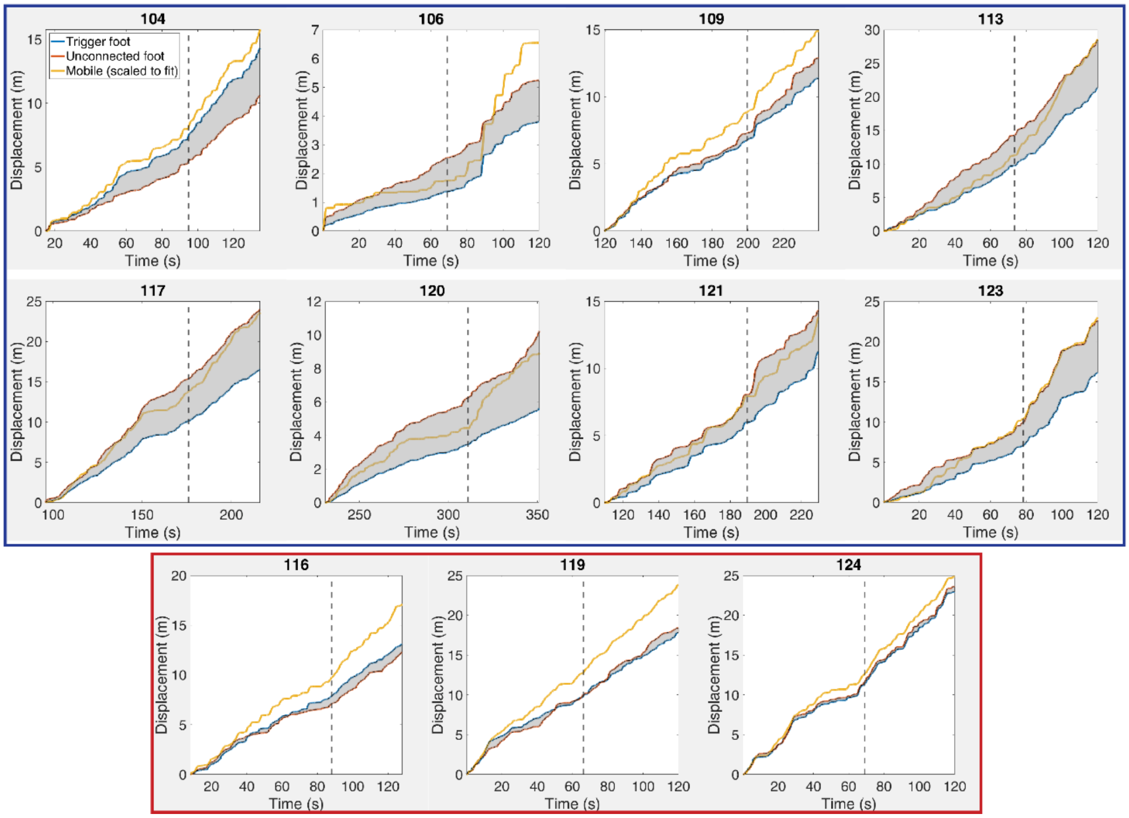

Fig. S3. Changing coordination within the baby's body and between baby and mobile surrounding the critical point. Plots of displacement of infants' feet and of the mobile in the tethered phase show inter-foot coordination (grey shading) surrounding the critical point. Greater area shaded in grey indicates weaker coordination between the feet. Dramatic changes in coordination between the feet surrounding the critical point are found only in infants who met the 150% cut-off (those in the blue lined outer box). For comparison, see a sample of infants who did not meet the cut-off in the box lined in red.

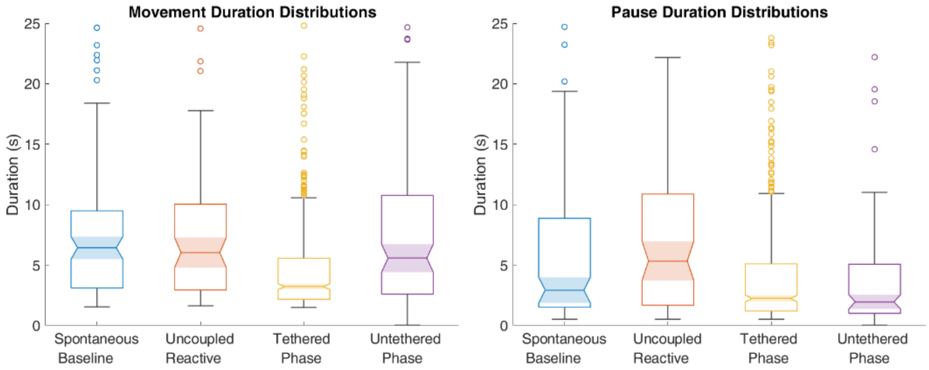

Fig. S4. Movement & pause durations change across context. Distributions of movement and pause durations for all 16 infants are plotted in the interval [0,25 sec.]. Both movements and pauses were generally quite short during tethering (movement median duration = 3.21s; pause median duration = 2.23s).

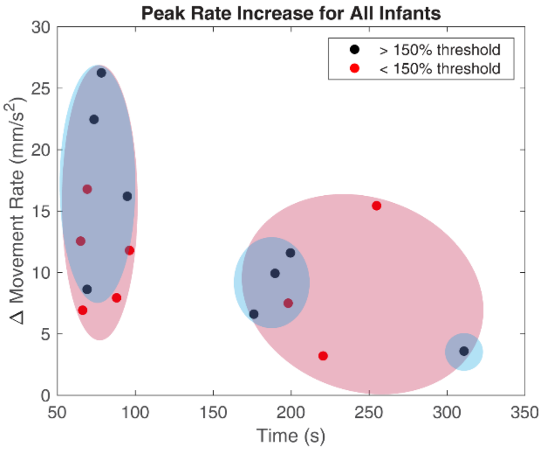

Fig. S5. Peak rate increase during tethering. Magnitude versus timing of peak Δ movement rate plotted for all infants (infants who increased activity ≥150% during tethering are marked in black while others are marked in red). K-means analysis of infants who exceed threshold suggests three distinct phenotypic patterns (blue ellipses, Fig. 3b), whereas k-means of all infants partition into two dynamical groups (pink ellipses). Clustering suggests different paths and timelines for agentive discovery.
